# Maculopapular eruption and fever due to lamotrigine followed by subsiding flare-ups

**DOI:** 10.1186/2045-7022-5-S1-P13

**Published:** 2015-03-11

**Authors:** Anna Valerieva, Maria Staevska, Vasil Dimitrov, Todor Popov

**Affiliations:** 1 Medical University - Sofia, Clinical Centre of Allergology, Clinic of Allergy and Asthma, Sofia, Bulgaria

## Introduction

Lamotrigine (LTG), an aromatic antiepileptic drug, is mainly used to manage epilepsy and bipolar / mood disorders. Skin rashes are the most common adverse reaction to this drug that typically develop in the first 8 weeks of treatment.

## Case presentation

A 27-year-old Caucasian woman treated with LTG 25 mg PO for a depressive episode was hospitalized in our allergy clinic with highly pruritic maculopapular eruption (MPE), affecting her abdomen, chest, back and forearms, which had started 2 days earlier along with fever of 37.5ºC. A well-defined red dermographism and tenderness of the skin was observed. She had been prescribed LTG 8 days before the symptoms commenced. A LTG-induced drug rash was suspected and the offending drug was therefore withdrawn.

The patient was prescribed methylprednisolone 60 mg/d IV, bilastine 2x20 mg/d PO, and chloropyramine 25 mg IM in the evening. Topical skin care with emollients was started, as well.

All blood and urine laboratory tests were within reference ranges except for the hsCRP of 9.6 mg/l (reference values up to 5 mg/l). Abdominal ultrasonography was performed and was unremarkable. The lesions subsided 7 days after starting the treatment. The patient was discharged with a prescription for methylprednisolone 20 mg/d PO, bilastine 20 mg/d PO and topical emollient skin care.

13 days later a new flare-up of MPE occurred. Again, laboratory tests were normal. The patient was prescribed methylprednisolone 40 mg/d IV, bilastine 2x20 mg/d PO and emollients. The eruption sustained for 3 weeks with frequent flare-ups. The corticosteroids’ regimen was adapted promptly with step downs and ups in order to maintain the severity of the symptoms. Antihistamines and emollients helped releive the subjective symptoms.

## Conclusion

MPE due to LTG is a common drug-induced hypersensitivity reaction. This reaction is generally mild. However, reactions should be monitored closely as they can be refractory and relapsing. This case presentation provides evidence that challenge tests should be performed at least 2 months after the discontinuation of the suspected drug, for there is a risk of false-positive reactions due to subsiding flare-ups.

## Consent

Written informed consent was obtained from the patient for publication of this abstract and any accompanying images. A copy of the written consent is available for review by the Editor of this journal.

